# Editorial: Emerging Micro- and Nanotechnologies for Medical and Pharmacological Applications

**DOI:** 10.3389/fphar.2021.648749

**Published:** 2021-02-22

**Authors:** Yufen Xiao, Jun Chen, Chao Wang, Jianxun Ding, Wei Tao

**Affiliations:** ^1^ Center for Nanomedicine and Department of Anesthesiology, Brigham and Women’s Hospital, Harvard Medical School, Boston, MA, United States; ^2^ Department of Bioengineering, University of California, Los Angeles, Los Angeles, CA, United States; ^3^ Jiangsu Key Laboratory for Carbon-Based Functional Materials and Devices, Institute of Functional Nano & Soft Materials (FUNSOM), Soochow University, Suzhou, China; ^4^ Key Laboratory of Polymer Ecomaterials, Changchun Institute of Applied Chemistry, Chinese Academy of Sciences, Changchun, China

**Keywords:** bioengineering, microtechnology, nanotechnology, pharmaceutics, controlled drug delivery, biomedical application

New features may emerge when a bulky material was engineered into micro-/nanoscale, which could be harnessed to bring innovations to the fields of medicine and pharmacology (Su et al.), e.g., targeted drug delivery by micro- and nanotechnologies to specifically treat human diseases (Ma et al.), like cancer (Wang et al.) and diabetes (Xiao et al.). This invited Research Topic contains 25 articles, including 15 original research papers, 9 review articles, and 1 minireview article, contributed by 199 researchers worldwide (Total views: 48,798; as of February 5, 2021), which covers a wide-range of research topics in the fields of medicine and pharmacology, including cancer diagnostics and therapy (Kim et al.), tissue engineering (Zhang et al.), diabetes treatment (Xu et al.), and others (Fan et al.).

Cancer therapies have been evolving with advances in oncology. This invited special issue covers both reviews and original research articles varying from chemodynamic therapy (CDT) and photothermal therapy (PTT) to immunotherapy. Among them, micro- and nanoparticles formed by polymers were meticulously designed and prepared. US Food and Drug Administration (FDA) approved biodegradable polymers, such as poly(ethylene glycol) (PEG), poly(lactide-*co*-glycolide) (PLGA), chitosan (CS), polylactide (PLA), and poly(ε-caprolactone) (PCL), are widely used to be self-assembled into nanoparticles. Among them, PEG, PLA, and CS can perform as hydrophilic moiety, while PLGA and PCL are hydrophobic chains. For instance, Li and co-workers developed PEG-PLGA micelle for loading sorafenib and all-trans retinoic acid to synergistically inhibit the growth of thyroid cancer *via* CDT. Injectable poly(ethylene glycol)-poly(L-valine) (PEG-PLV) hydrogel (Shan et al.) and PLGA microparticle (Zheng et al.) were developed for the sustained release of chemotherapeutics. Natural materials, like CS and hyaluronic acid (HA) nanoparticles, are also good candidates for delivering hydrophobic drugs, such as 10-hydroxycamptothecin (HCPT). HCPT-loaded CS nanoparticle developed by Guo et al. showed enhanced penetration in tumor sites and improved chemotherapy of melanoma. By applying CD55 ligand peptide, CS nanoparticle showed targeted antitumor efficacy (Liu et al.). HA was capable of encapsulating doxorubicin and cisplatin as a pH-responsive nanocarrier for the inhibition of breast cancer (Yu et al.). In order to achieve better penetration of drugs, cell-penetration peptides (CPPs) were introduced (Xie et al.). By combination with CGKRK peptide, paclitaxel prodrug nanoparticle demonstrated enhanced penetration against the blood-brain barrier (BBB) (Lv et al.). In addition to CPP modification, the shape also played an important role in nanoparticle penetration (Cao et al.). The ultra-small nanoparticle exhibited the ability of crossing BBB with enhanced cell uptake, demonstrating potential in tumor therapeutics.

Apart from polymer materials, inorganic biomaterials and cell technology are also included in this special issue. Among them, two-dimensional (2D) nanosheets attracted the most attention due to their large surface area for high loading of drugs and excellent photothermal conversion efficiency (Kim et al.). Mesoporous silica nanoparticles also benefited from a large surface-to-volume ratio in delivering a gene to ameliorate metabolic disorders (Tao et al.). Magnetic nanoparticles held the advantages in cancer theranostic due to their role in generating reactive oxygen species (ROS) for CDT and relaxation rate for diagnosis (Guo et al.). Silver nanoparticle was generally considered as an antibacterial material (Wang et al.). Cell technology is rapidly developed because of the biocompatibility and multi-functionality of the cell. Peripheral blood-derived stem cells were capable of cartilage repair regeneration (Chen et al.). Dendritic cells were responsible for immune responses, which play a significant role in cancer immunotherapy (Qian et al.). Stem cells were progenitor cells capable of differentiating into various cell lineages, such as islet β cells to mimic the artificial pancreas (Xu et al.). By coating with cell membranes, nanocarriers could obtain the ability to escape from immune response and accurately reach targeted sites for cancer therapy (Li et al.).

In addition to the drug delivery system, tissue engineering is another crucial application of microtechnology. Several reviews summarized different platforms for tissue engineering. For example, hydrogels could be displayed as scaffolds to optimize the microenvironment for bone regeneration (Zhang et al.). Besides, hydrogels were also carriers for gene delivery to selectively upregulate or knockdown target genes for osteochondral regeneration (Yan et al.). One review article discussed the unique approaches to aged skeletal muscle loss. Another review presented by Fan et al. summarized the recent progress in 3D printing technology, one of the most exciting and precise manufacturing technologies. Original research covering the vital aspect of engineering is also included. For instance, 3D PCL scaffolds developed by Zhou et al. exhibited enhanced proliferation and adhesion of cells. The advanced surgical suture could be obtained by polyurethane fiber with antibacterial and shape memory properties (Zhou et al.). Jin et al. developed gene-loaded scaffold with adipose-derived stem cells for bladder repair. The scaffold is capable of drug loading to reach sustainable and controllable drug release at specific sites during repair (Shan et al.).

Overall, the current special issue reports a diverse intersection of micro- and nanotechnologies that showed promise potential in engineering materials for biomedical and pharmacological applications. Such an interdisciplinary investigation is building a bridge between fundamental material innovation and clinical medical translation, greatly benefiting fields of micro-/nanotechnology, bioengineering, and pharmaceutics.

